# Connecting Social Determinants of Health to Cancer Care: Access to Cancer Care to Patient Experiences of Older Adults

**DOI:** 10.1093/geroni/igaf122.1208

**Published:** 2025-12-31

**Authors:** Mackenzie Fowler

## Abstract

Social determinants of health (SDOH) have emerged as important predictors of outcomes in cancer. SDOH are the “conditions in which people are born, grow, learn, work, play, live, and age, and the broader set of structural factors shaping the conditions of daily life”. These are generally described in five domains: economic stability, education access/quality, healthcare access/quality, neighborhood/built environment, social/community context. SDOH span all levels of the social ecological framework from health policy to the individual. However, limited evidence exists examining the relationship between SDOH among older adults with cancer. Our symposium brings together researchers to evaluate SDOH across domains and levels of influence and their relationship with outcomes among older adults with cancer. First, Dahal will present the policy level of influence and healthcare access/quality domain by describing access to colon cancer care for vulnerable Medicare-Medicaid dual enrollees, specifically plan enrollment at diagnosis. Second, Fowler will present the community level and neighborhood/built environment domain describing the adjusted association between neighborhood-level social vulnerability and its differential effect on cancer survival among older versus younger adults. Finally, Swaim will discuss the individual level and social/community context domain to understand how daily experiences may fluctuate within a person day-to-day. Understanding how SDOH across domains and levels of influence affect outcomes among older adults with cancer will help facilitate opportunities for intervention specific to the unique needs of this population.

